# Adaptive MR-Guided Stereotactic Radiotherapy is Beneficial for Ablative Treatment of Lung Tumors in High-Risk Locations

**DOI:** 10.3389/fonc.2021.757031

**Published:** 2022-01-11

**Authors:** Sebastian Regnery, Carolin Buchele, Fabian Weykamp, Moritz Pohl, Philipp Hoegen, Tanja Eichkorn, Thomas Held, Jonas Ristau, Carolin Rippke, Laila König, Michael Thomas, Hauke Winter, Sebastian Adeberg, Jürgen Debus, Sebastian Klüter, Juliane Hörner-Rieber

**Affiliations:** ^1^ Department of Radiation Oncology, Heidelberg University Hospital, Heidelberg, Germany; ^2^ National Center for Radiation Oncology, Heidelberg Institute for Radiation Oncology, Heidelberg, Germany; ^3^ National Center for Tumor diseases, Heidelberg, Germany; ^4^ Heidelberg Ion-Beam Therapy Center, Department of Radiation Oncology, Heidelberg University Hospital, Heidelberg, Germany; ^5^ Clinical Cooperation Unit Radiation Oncology, German Cancer Research Center, Heidelberg, Germany; ^6^ Institute of Medical Biometry, University of Heidelberg, Heidelberg, Germany; ^7^ Department of Thoracic Oncology, Thoraxklinik at Heidelberg University Hospital, Heidelberg, Germany; ^8^ Translational Lung Research Center Heidelberg, Member of the German Center for Lung Research, Heidelberg, Germany; ^9^ Department of Thoracic Surgery, Thoraxklinik at Heidelberg University Hospital, Heidelberg, Germany

**Keywords:** stereotactic body radiotherapy, image-guidance, radiotherapy, pulmonary cancer, magnetic resonance imaging, MR-guided radiotherapy

## Abstract

**Purpose:**

To explore the benefit of adaptive magnetic resonance-guided stereotactic body radiotherapy (MRgSBRT) for treatment of lung tumors in different locations with a focus on ultracentral lung tumors (ULT).

**Patients & Methods:**

A prospective cohort of 21 patients with 23 primary and secondary lung tumors was analyzed. Tumors were located peripherally (N = 10), centrally (N = 2) and ultracentrally (N = 11, planning target volume (PTV) overlap with proximal bronchi, esophagus and/or pulmonary artery). All patients received MRgSBRT with gated dose delivery and risk-adapted fractionation. Before each fraction, the baseline plan was recalculated on the anatomy of the day (predicted plan). Plan adaptation was performed in 154/165 fractions (93.3%). Comparison of dose characteristics between predicted and adapted plans employed descriptive statistics and Bayesian linear multilevel models. The posterior distributions resulting from the Bayesian models are presented by the mean together with the corresponding 95% compatibility interval (CI).

**Results:**

Plan adaptation decreased the proportion of fractions with violated planning objectives from 94% (predicted plans) to 17% (adapted plans). In most cases, inadequate PTV coverage was remedied (predicted: 86%, adapted: 13%), corresponding to a moderate increase of PTV coverage (mean +6.3%, 95% CI: [5.3–7.4%]) and biologically effective PTV doses (BED_10_) (BED_min_: +9.0 Gy [6.7–11.3 Gy], BED_mean_: +1.4 Gy [0.8–2.1 Gy]). This benefit was smaller in larger tumors (−0.1%/10 cm³ PTV [−0.2 to −0.02%/10 cm³ PTV]) and ULT (−2.0% [−3.1 to −0.9%]). Occurrence of exceeded maximum doses inside the PTV (predicted: 21%, adapted: 4%) and violations of OAR constraints (predicted: 12%, adapted: 1%, OR: 0.14 [0.04–0.44]) was effectively reduced. OAR constraint violations almost exclusively occurred if the PTV had touched the corresponding OAR in the baseline plan (18/19, 95%).

**Conclusion:**

Adaptive MRgSBRT is highly recommendable for ablative treatment of lung tumors whose PTV initially contacts a sensitive OAR, such as ULT. Here, plan adaptation protects the OAR while maintaining best-possible PTV coverage.

## Introduction

Stereotactic body radiotherapy (SBRT) represents the standard treatment for inoperable early-stage NSCLC (1) and enables good local control for pulmonary oligometastases (2–4). Current state-of-the-art SBRT techniques mainly rely on CT-based image-guided radiotherapy (IGRT) (5). This usually includes 4D-CT-derived internal target volume (ITV) approaches, where the ITV encompasses the whole tumor trajectory during breathing (5–7). In general, outcomes of pulmonary SBRT using state-of-the-art techniques are favorable, with high local control rates after application of ablative biologically effective doses (α/β = 10, BED_10_) >100 Gy (8, 9) and low treatment-related toxicity (2, 4, 10, 11).

Recent advances in magnetic resonance-guided SBRT (MRgSBRT) offer high-precision treatment for lung tumors on hybrid MR-linac systems. Essentially, cineMR-imaging during irradiation enables constant visualization of the intrafractional tumor motion together with gated dose delivery (12, 13). Thus, gating replaces ITV approaches, which significantly reduces the irradiated lung volumes (6, 14). Additionally, MRgSBRT offers the opportunity of online plan adaptation to correct for interfractional changes in thoracic anatomy. Here, the baseline plan is recalculated on a daily pre-treatment MRI and can be adapted to the anatomy of the day with the patient lying on the couch, if necessary (12). Previous investigations of adaptive MRgSBRT of lung tumors have demonstrated its clinical feasibility and suggested dosimetric benefits compared to non-adaptive SBRT and also favorable clinical outcomes (7, 15, 16). However, MRgSBRT with gated dose delivery already represents an elaborate innovation which becomes significantly more labor-intensive and time-consuming with additional online plan adaptation (17). Given the already favorable outcomes of standard non-adaptive pulmonary SBRT, targeted patient selection based on evidence of meaningful benefits is key to the reasonable clinical use of adaptive MRgSBRT.

Current state-of-the art SBRT techniques face major challenges when ablative biologically effective doses >100 Gy should be applied to lung tumors that lie close to sensitive organs-at-risk (OAR), such as central lung tumors (18–20). Ultracentral lung tumors (ULT), which immediately touch the proximal bronchial tree (PBT), esophagus or pulmonary artery, are at especially high risk for severe toxicity after SBRT, so that local tumor control must be weighed against toxicity (19–22). Only recently, the first prospective data on SBRT of ULT was released from the HILUS trial and demonstrated high rates of severe toxicity after application of 8 × 7 Gy to the ULT (23). In case of such “high-risk” lung tumor locations, adaptive MRgSBRT could significantly improve the therapeutic ratio. Previous series indeed suggested a meaningful benefit of adaptive MRgSBRT in central and ultracentral lung tumors (15, 16, 24). Conversely, SBRT of peripheral lung tumors far from sensitive OAR might primarily benefit from gated dose delivery, but not as much from online plan adaptation (7).

We hypothesize that an ultracentral lung tumor location predicts a clinically meaningful benefit of online plan adaptation and thus represents a useful criterion to select patients for adaptive MRgSBRT. Therefore, we aim to explore the dosimetric benefits and clinical feasibility of adaptive MRgSBRT for different lung tumor locations in a prospective patient cohort with a focus on ULT.

## Patients and Methods

### Patient Characteristics

This analysis comprises 21 patients from two consecutive prospective registries who received pulmonary MRgSBRT with gated dose delivery and daily plan adaptation between February 2020 and January 2021. In total, 23 primary (N = 10) and secondary (N = 13) pulmonary lesions were treated. Eleven lesions were ultracentrally located, defined as an overlap of the PTV with the PBT, esophagus and/or pulmonary artery. Two lesions were centrally located according to the RTOG 0813 definition [lung tumors within 2 cm from the PBT or touching mediastinal or pericardial pleura (25)] and ten lesions were peripherally located. Patient and lesion characteristics are summarized in **Table 1**. Patients frequently suffered from several comorbidities [Charlson Comorbidity Index (CCI), excluding oncological diagnosis: median 3 (2–5)] and reduced pulmonary function [forced expiratory volume in the first second (FEV1s in % of the predicted value): 68.0% (55.1–88.4%)].

**Table 1:** Patient and treatment characteristics.

Patients (N = 21)		
	Median	IQR
**Age**	65.4	59.1–75.0
**CCI**	3	2–5
**KPI [%]**	80	80–90
**FEV1s [% of predicted]**	68.0	55.1–88.4
	**N**	**%**
**Sex**		
** Male**	15	71.4
** Female**	6	28.6
**COPD**		
** Grade I**	2	9.5
** Grade II**	4	19.1
** Grade III**	2	9.5
** None**	13	61.9
**Smoking History**		
** Yes (≥25 py)**	9	42.9
** No**	9	42.9
** N/A**	3	14.3
**Tumor Entity**		
** NSCLC**	11	52.4
** Non-pulmonary**	10	47.6
**Treatment Situation**		
** Early-stage NSCLC**	6	28.6
** Local relapsing NSCLC**	1	4.8
** Oligoprogression**	14	66.7
**Lesions (N = 23)**		
	**Median**	**IQR**
**GTV Size [cm³]**	6.98	3.7–24.6
**CTV Size [cm³]**	12.80	7.1–38.9
**PTV Size [cm³]**	22.80	15.4–56.5
	**N**	**%**
**Location**		
** Peripheral**	10	43.5
** Central**	2	8.7
** Ultracentral**	11	47.8
** PBT**	6	
** Esophagus**	5	
** PA**	1	
**Fractionation**		
** 3 × 9–15 Gy**	3	13.0
** 5 × 10 Gy**	5	21.7
** 5 × 6 Gy, 6–8 × 5 Gy**	3	13.0
** 8 × 7.5 Gy**	5	21.7
** 10 × 5–6 Gy**	6	26.1
** 12 × 5 Gy**	1	4.3

N, absolute number; IQR, interquartile range; CCI, Charlson Comorbidity Index (excluding oncological diagnosis); KPI, Karnofsky Performance Index; FEV1s, forced expiratory volume in the first second; COPD, chronic obstructive pulmonary disease; py, pack years; N/A, not available; NSCLC, non-small cell lung cancer; GTV, gross tumor volume; CTV, clinical target volume; PTV, planning target volume; PBT, proximal bronchial tree; PA, pulmonary artery; Gy, Gray.

### Treatment Simulation and Planning

Patients were treated on a 0.35 Tesla (T) MRIdian Linac^®^ system with a 6-megavolt linear accelerator (ViewRay Inc., Oakwood, USA) (12, 13). Pre-treatment simulations have already been described earlier (26). Briefly, patients were immobilized and received simulation at the MR-linac including 3D MRI in deep inspiration breath-hold (DIBH) (resolution: 1.5 × 1.5 mm^2^, breath-hold: 17–25 s with a slice thickness of 3 mm) and also 2D cineMRI (resolution: 0.243 × 0.70 cm^2^, 4–8 frames/s) (12). Immediately afterwards (usually within 1 h from MR-simulation), patients underwent thoracic planning CT (Siemens SOMATOM Confidence^®^, Siemens Healthineers, Germany) with identical immobilization. The gross tumor volume (GTV) encompassed areas of macroscopic tumor spread on MR- and CT-imaging and was isotropically expanded by 2 mm to obtain the clinical target volume (CTV), thereby respecting anatomical borders (adjacent OAR), and by another 3 mm to obtain the PTV according to our institutional standards.

### Dose Prescription

SBRT was delivered as step-and-shoot intensity modulated radiotherapy (IMRT) as described previously (26). The most commonly applied fractionation schemes were 10 × 5–6 Gy to ultracentral tumors, 8 × 7.5 Gy to central tumors and 5 × 10 Gy to peripheral tumors (**Table 1**, **Supplementary Table 1**). Generally, RT plans were optimized to yield a 95% conformal coverage of the PTV with the prescription dose (PD) (target dose) with maximum doses at 125%. For a fractionation of 3 × 15 Gy, 150% of the PD was chosen as maximum dose. Three cases of ULT required modifications to comply with OAR dose constraints. Here, RT plans were prescribed to the median dose aiming at 95% coverage of the PTV by 95% of the PD (target dose) and less than 1–2% of the PTV was allowed to exceed 107% of the PD. Dose constraints for different OARs and fractionations are given in **Supplementary Table 2** (5, 16, 27). Violation of dose constraints always triggered online adaptation. Furthermore, priority was given to OAR constraints, so that target coverage was compromised if required.

### Plan Adaptation

After patient placement and immobilization, a 3D MRI was performed in DIBH and was rigidly registered to the planning MRI based on the GTV contours. OAR contours and pre-treatment CT-imaging were deformably registered to this MRI of the day using a vendor-supplied algorithm. Subsequently, GTV contours were adapted by the treating physician and OAR contours were edited in a region expanding 1 cm in cranio-caudal direction and 3 cm in all other directions from the PTV (PTV_expand_) on the MRI of the day (28). The baseline plan was applied to this anatomy of the day, yielding the predicted plan. Based on the predicted plan, the treating physician could initiate plan re-optimization using the same planning objectives and beam parameters as in the baseline plan, which led to the adapted plan. Plan re-optimization was mandatory if planning objectives were violated. Before delivery of the adapted plan, on-table quality assurance (QA) was performed including vendor-supplied secondary dose calculation together with an in-house developed software solution to assess contour integrity, target volume expansion and fluence modulation complexity (29).

### Gated Dose Delivery

SBRT was delivered with real-time MR gating as described previously (26). Briefly, a region of interest (ROI) was selected on 2D cineMRI (e.g., a tumor (sub)region) and a gating boundary was defined by adding a 3 mm margin. During irradiation, 2D cineMRI was constantly active to verify location of the ROI inside this boundary on a single sagittal slice. A small percentage of the ROI was allowed outside the boundary (in general threshold-ROI% = 3%) to account for image noise and registration uncertainties. The radiation beam was automatically turned off when this threshold was exceeded.

### Statistical Analysis

Statistical analysis was performed using R with the `brms` package (30) in version 4.0.3. The predicted and adapted plan was available for all fractions where plan adaptation was performed (N = 154, 93.3%). The underlying observations (N = 308) were clustered in the 23 irradiated lesions, so that observations from fractions applied to the same irradiated lesions could not be considered independent. This restricted the number of independent observations and thus the feasibility of a frequentist parameter estimation adjusting for multiple covariates. Instead, we chose a Bayesian approach, which is feasible for any number of observations. We used Bayesian linear multilevel models (LMM) for assessing the correlation between different response variables with plan adaption, tumor location, and PTV size. This allowed us to explore the effect of plan adaption on several response variables of interest while adjusting for tumor location, PTV size, and potential interactions. The LMM incorporated two group level effects as varying intercepts with default priors for the standard deviations [half Student t prior with three degrees of freedom and a scale parameter ≥10 (30)], namely one identifier for each irradiated lesion and one identifier for each single fraction.

Plan adaptation and ultracentral tumor location and their interaction were incorporated as binary population level effects. The target volume size and its interaction with plan adaptation were incorporated as continuous population level effects. Weakly informative priors were chosen for all population level effects. LMM with PTV, CTV, and GTV coverage by the target dose and also the minimum BED_10_ (BED_min_) and the mean BED_10_ (BED_mean_) inside the PTV, CTV, and GTV as response variables served to assess target volume coverage and dose. The BED_10_ was calculated assuming an α/β ratio of 10 inside the tumor according to the linear-quadratic formula:


BEDαβ=n·d·(1+dα/β)

Additionally, we employed a LMM with the percentage of PTV exceeding the dose maximum as response variable to evaluate target overdoses. The general appearance of these LMM was as follows:


$$Response∼Normal(R_{Mean,}R_{STD})$$

$$R_{Mean}=a\cdot adaptation+l\cdot location+i\cdot location\cdot adaptation+s\cdot size+j\cdot size\cdot adaptation+intercept+\alpha \cdot ID_{lesion}+\beta \cdot ID_{fraction}$$

*a, l, s, i, j*: parameters of population-level effects to be estimated

*ID_lesion_, ID_fraction_*: identifiers (design matrices) for the irradiated lesion and fraction

*α, β*: parameter vectors of group-level effects to be estimated

To assess violation of OAR constraints, a logistic LMM with OAR constraint violation as binary response variable and plan adaptation, tumor location and PTV size as population level effects was used.


Violation ∼ Binomial(1,p)

$$logit(p)=a\cdot adaptation+l\cdot location+s\cdot size+intercept+a\cdot ID_{lesion}+\beta \cdot ID_{fraction}$$

Finally, PTV size was regressed on the fraction number to evaluate longitudinal changes of PTV size.


$$Size∼Normal(Size_{Mean},Size_{STD})$$

$$Size_{Mean}=n\cdot number_{fraction}+intercept+\;+\alpha \cdot ID_{lesion}$$

Posterior distributions were derived from Markov Chain Monte Carlo simulations with 8 chains, each employing 8,000 iterations with 3,000 warm-up samples. $\hat{R}$ -values were calculated to evaluate convergence of the different chains towards the target posterior distribution and thus the validity of approximation. All $\hat{R}$ -values were ≤1.01, which support the convergence of the chains (**Supplementary Tables 3–14**). Furthermore, we confirmed that the overall results remained the same when priors were varied. The robustness of the parameter distributions is described by the 95% compatibility interval, which contains 95% of the probability mass of the respective posterior distribution.

### Ethics Statement

The two underlying prospective registries were initiated and maintained in accordance with the declaration of Helsinki and received local ethics board approval. Written informed consent was obtained from all patients before inclusion.

## Results

### Planning Target Violations

**Figure 1:**
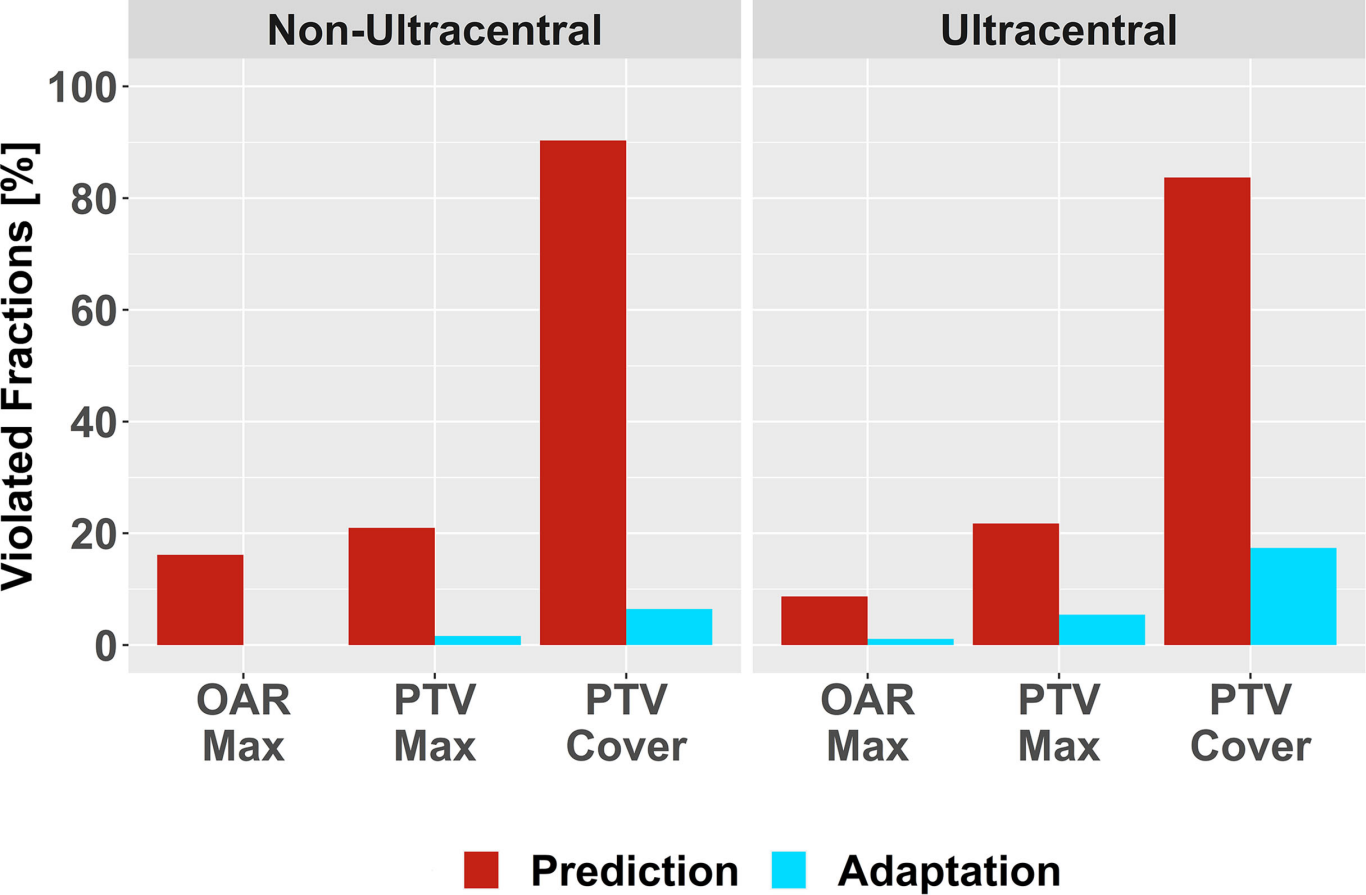
Violation of Planning Objectives. Relative number of violated planning objectives before (red) and after plan adaptation (blue) for non-ultracentral and ultracentral lung tumors: exceeded maximum dose inside organs-at-risk (OAR Max) or inside the planning target volumes (PTV Max); inadequate PTV coverage (PTV Cover).

A total of 165 SBRT fractions were delivered and daily plan adaptation was performed in 154 fractions (93.3%). In 145 fractions that underwent plan adaptation (94.2%), at least one planning objective was violated inside the predicted plan as main trigger of plan adaptation. Inadequate PTV coverage (133 fractions, 86.4%) represented the most frequent violation, followed by exceeded maximum dose inside the PTV (33 fractions, 21.4%) and exceeded maximum dose inside OAR (18 fractions, 11.7%). Plan adaptation strongly reduced the amount of planning objective violations to 26 fractions (16.9%). Again, residual violations were most frequently due to inadequate PTV coverage (20 fractions, 13.0%), followed by exceeded maximum dose inside the PTV (6 fractions, 3.9%) and exceeded maximum dose inside OAR (1 fraction, 0.6%). Descriptive analysis revealed that residual violations mostly occurred in ultracentral lesions and primarily due to inadequate PTV coverage (**Figure 1**).

### Target Volume Coverage

**Figure 2:**
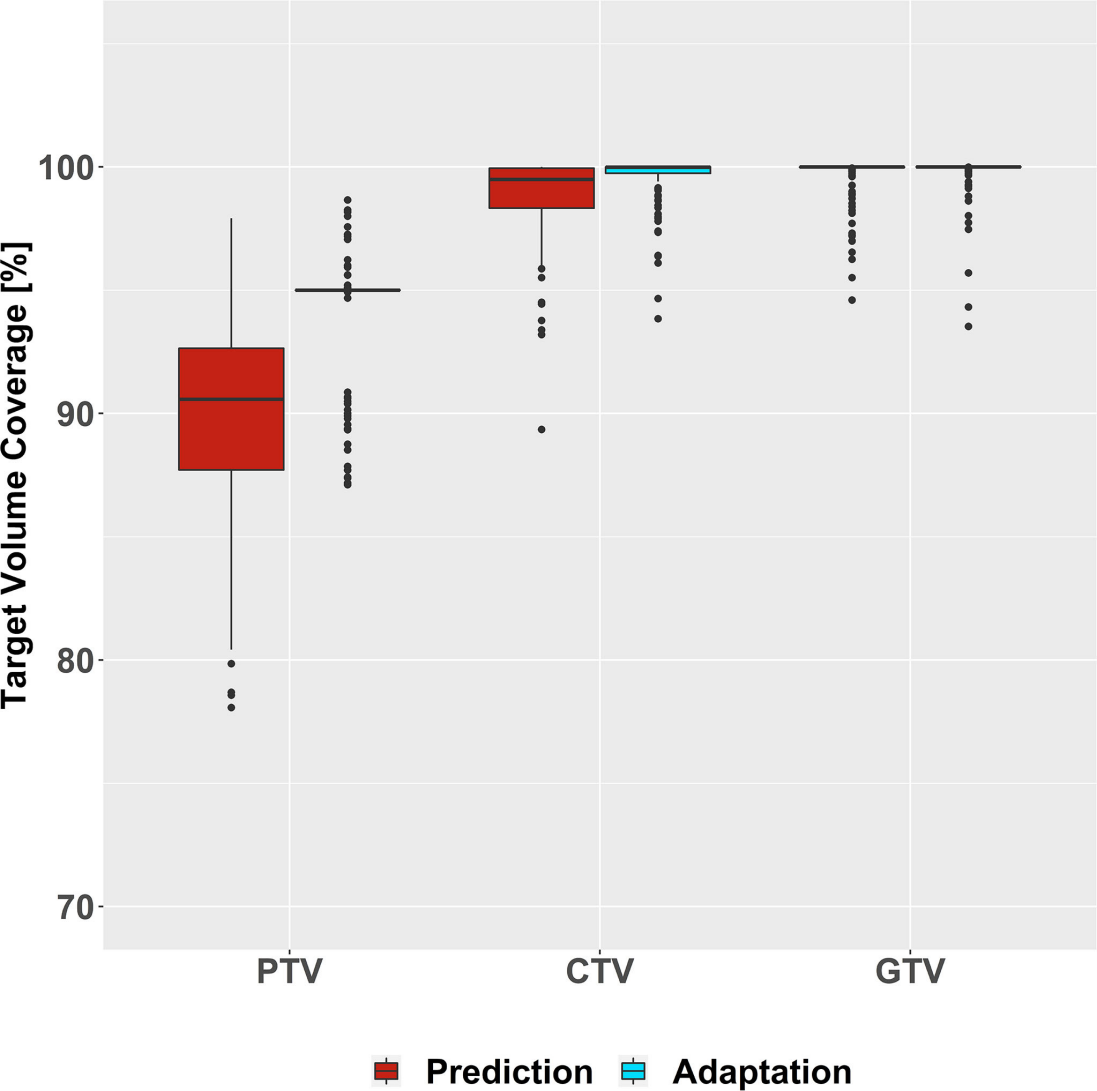
Coverage of Target Volumes. All target volume coverages are given in percent of the target covered by the target dose before (red) and after (blue) plan adaptation.

A descriptive analysis demonstrated that plan adaptation increased PTV coverage (predicted: median: 90.6%, interquartile range [87.7–92.6%]; adapted: 95.0% [95.0–95.0%]), which translated to a slightly higher CTV coverage after plan adaptation (predicted: 99.5% [98.3–100%]; adapted: 100% [99.7–100%]). GTV coverage remained similarly high before and after plan adaptation (predicted: 100% [100–100%], adapted: 100% [100–100%]) (**Figure 2**). The LMM of PTV coverage was compatible with an absolute 6.3% increase of PTV coverage due to plan adaptation (95% compatibility interval (95%-CI): [5.3–7.4%]). Moreover, PTV size and also ultracentral tumor location presented negative interactions with plan adaptation that are compatible with a decreased impact of plan adaptation on PTV coverage for larger tumors (plan adaptation × PTV size: −0.1%/10 cm³ PTV [−0.2 to −0.02%/10 cm³ PTV]) and ultracentral tumors (plan adaptation × ultracentral location: −2.0% [−3.1 to −0.9%]) (**Supplementary Table 3**, **Supplementary Figure 1**). There was a trend towards increased CTV coverage after plan adaptation (0.4% [−0.01 to 0.7%]), while GTV coverage was suggested to be similar before and after plan adaptation (−0.02% [−0.2 to 0.2%]) (**Supplementary Tables 4 and 5**).

The LMM of the BED_min_ were compatible with an increase due to plan adaptation that declined successively from PTV (9.0 Gy [6.7–11.3 Gy]) to CTV (6.5 Gy [4.5–8.5 Gy]) to GTV (2.9 Gy [1.2–4.5 Gy]) (**Supplementary Figure 2**, **Supplementary Tables 6–8**). Correspondingly, the LMM of the BED_mean_ were compatible with a slight increase due to plan adaptation inside the PTV (1.4 Gy [0.8–2.1 Gy]) and CTV (0.8 Gy [0.2–1.5 Gy]) and showed a positive trend inside the GTV (0.6 Gy [−0.1 to 1.2 Gy]) (**Supplementary Figure 3**, **Supplementary Tables 9–11**).

### Target Volume Overdose

**Figure 3:**
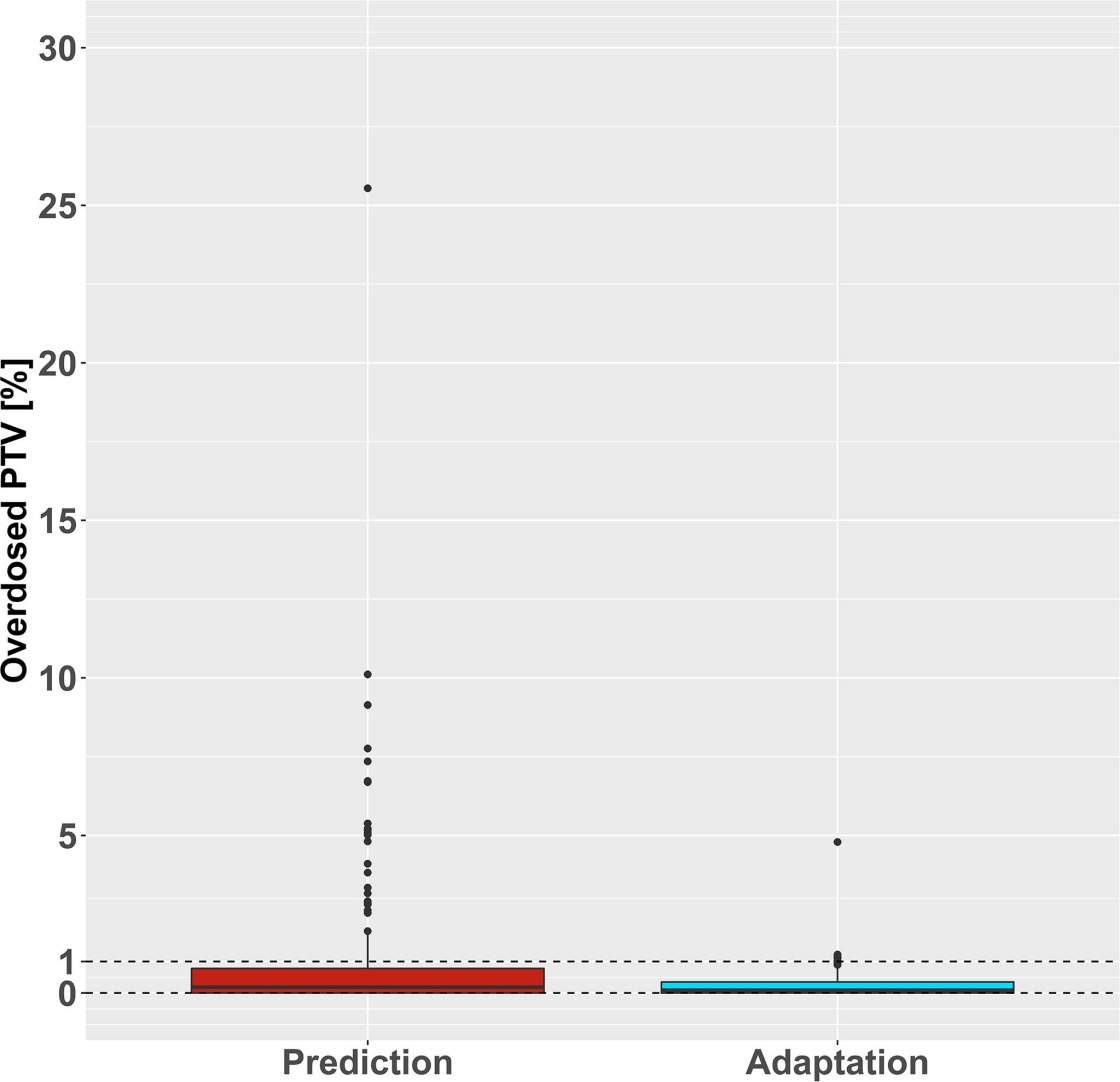
Target Volume Overdose. The relative planning target volume (PTV) that received a radiation dose above the dose maximum is shown before (red) and after (blue) plan adaptation. Generally, a maximum volume of 1% was allowed to exceed the maximum dose (dashed line).

PTV overdoses were measured by the percentage of PTV exceeding the maximum dose according to the planning objectives. The predicted plans showed increased proportions of PTV above the aimed maximum dose (median: 0.2%, range: 0–25.5%) compared to the adapted plans (median: 0.1%, range: 0–4.8%). Of the 33 predicted plans with an overdose inside the PTV, 12 exceeded the maximum dose in >5% of the PTV. Conversely, only six adapted plans exceeded the planning objective for the maximum PTV dose, all by <5% of the PTV (**Figure 3**). The LMM for PTV exceeding the maximum dose was compatible with a reduction of absolute −1% after plan adaptation (95% CI: [−1.7 to −0.2%]) but did not support an association of ultracentral tumor location or PTV size with PTV overdoses (**Supplementary Table 12**).

### OAR Constraints

**Figure 4:**
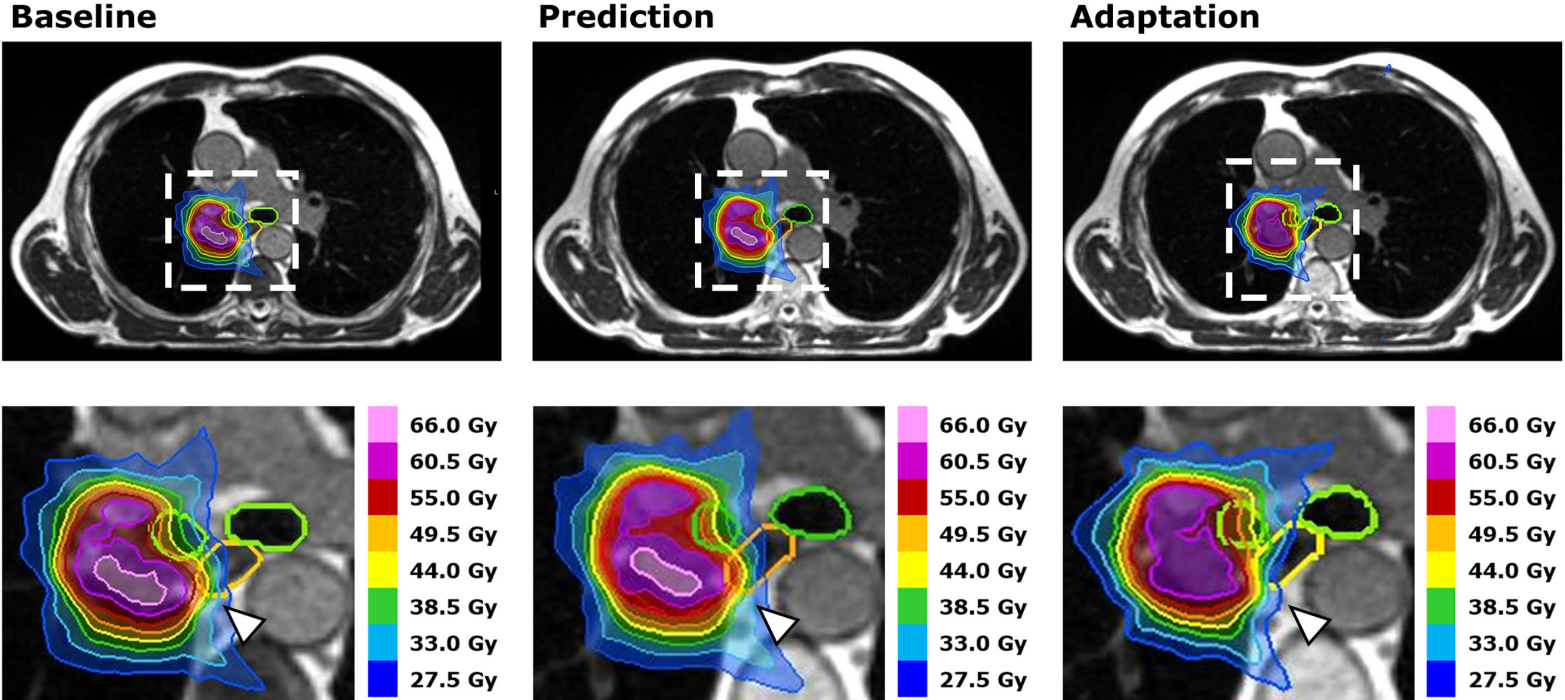
Case Study. Dose distributions for dose intensified SBRT (10 **×** 5.5 Gy) of an ultracentral lung tumor adjacent to the right main stem bronchus at baseline (left), after plan prediction (middle) and after plan adaptation (right). Segmentations of the proximal bronchial tree (green) and the esophagus (orange) demonstrate a shift of the esophagus into the high dose volume after plan prediction. This led to a violation of the esophageal dose constraint, which was remedied successfully by plan adaptation (white arrowheads).

**Figure 5:**
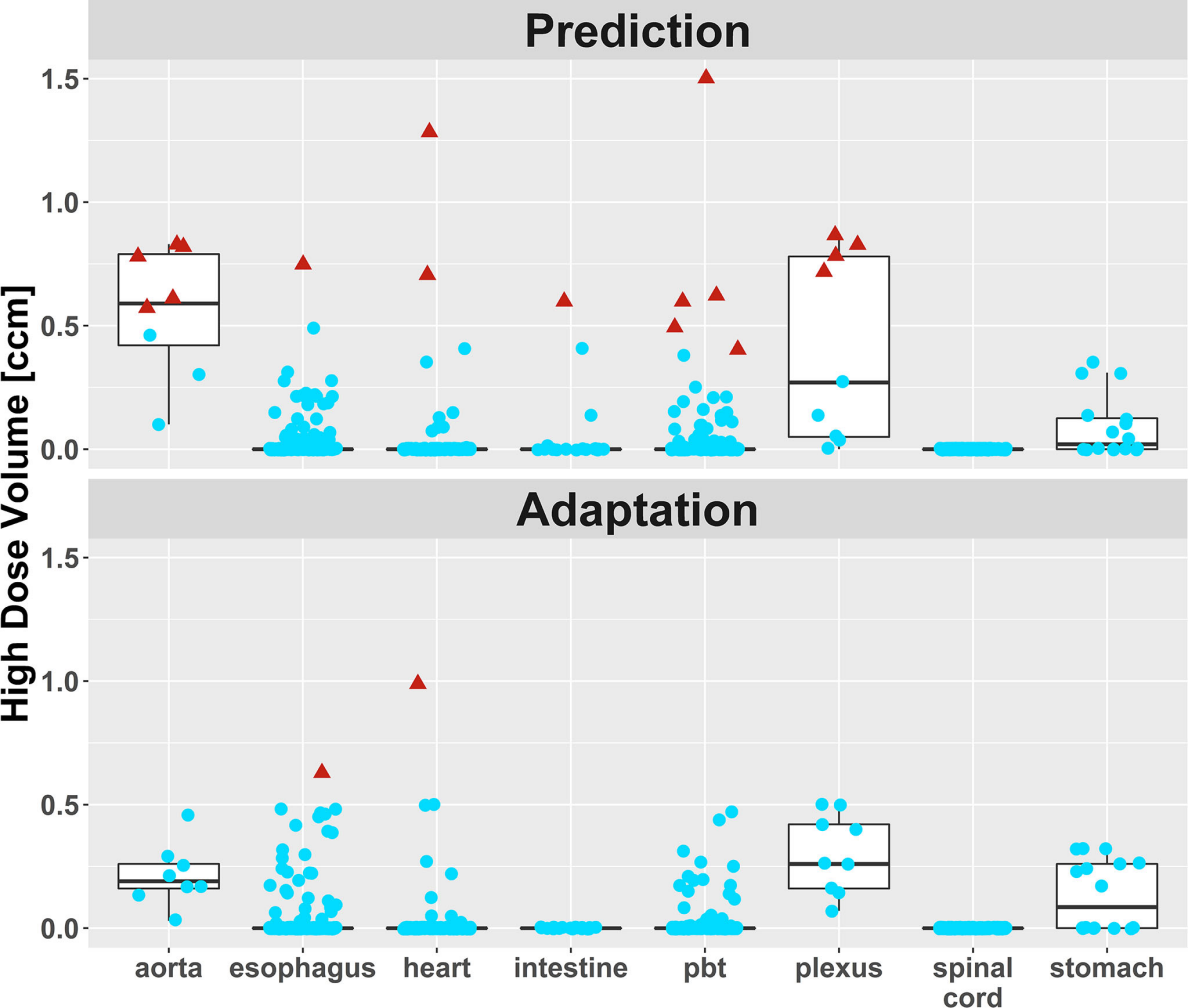
High dose volumes of different organs-at-risk (OAR). Blue dots: High dose volume lies below the planning objective (mostly <0.5 cm³). Red triangles: High dose volume exceeds the planning objective. There was one remaining fraction with two moderate OAR constraint violations in an ultracentral lung tumor, where a compromise between target volume coverage and dose inside the OAR was chosen. No more OAR constraint violations occurred in this patient.

Violations of OAR constraints were detected in 18 fractions of six different patients at plan prediction (**Figure 4**). Eight violations occurred in ULT and five violations occurred in central and peripheral tumors, respectively. In 17 fractions with violated OAR constraints (five out of six patients), the PTV had touched the corresponding OAR in the baseline plan (distance ≤1 voxel). The remaining case was the SBRT of a “peripheral” lung tumor above the right diaphragm that had moved close to the intestines due to different breath hold in one fraction. After plan adaptation, only one fraction with OAR constraint violations remained in one patient with an ULT (**Figure 5**, **Supplementary Table 13**). The logistic LMM was compatible with a reduction of OAR constraint violations after plan adaptation (OR = 0.14 [0.04–0.44]) but did not support an influence of ultracentral tumor location or PTV size on OAR constraint violations after plan adaptation (**Supplementary Table 14**).

### PTV Size Changes

Descriptive analysis showed a stable development of PTV size over the course of SBRT (**Supplementary Figure 4**). The LMM of PTV changes per fraction was compatible with a minor volume increase of 0.4 cm³ for each successive fraction (95% CI: [0.1–0.7 cm³/fraction]) (**Supplementary Table 15**).

### Feasibility

The mean times for recontouring, plan adaptation (including QA), irradiation (beam-on and beam-off), and total treatment were 14.8 ± (SD) 6.9, 12.9 ± 6.5, 15.3 ± 7.2, and 55.8 ± 12.8 min, respectively. Times for recontouring (ULT: 17.1 ± 6.6 min, non-ULT: 11.5 ± 5.9 min) and plan adaptation (ULT: 14.4 ± 7.0 min, non-ULT: 10.7 ± 5.1 min) and also total treatment times (ULT: 57.9 ± 12.7 min, non-ULT: 52.6 ± 12.4 min) tended to be higher for ULT, whereas irradiation times were smaller (ULT: 13.9 ± 7.6 min, non-ULT: 17.3 ± 6.2 min) (**Supplementary Figure 5**). Only one patient had to cancel daily plan adaptation because of increasing bone pain when lying still after seven of ten SBRT fractions. The remaining three fractions could be administered as MRgSBRT with gating using the baseline plan.

## Discussion

Together with previous reports (7, 15, 16, 31), our data underline the clinical feasibility of adaptive MRgSBRT for treatment of lung tumors, including, high-risk tumor locations and patients with reduced pulmonary function. However, plan adaptation is costly. Firstly, plan adaptation approximately doubled the treatment time in this cohort. The fact that treatment times are somewhat higher than reported previously (7, 15) could be explained by the inclusion of more ULT with longer recontouring and adaptation times due to proximity to various mediastinal OAR. Secondly, adaptive MRgSBRT is a labor-intensive technique, since one radiation oncologist and one medical physicist need to be present most of the time during treatment. Therefore, evidence-based criteria to identify patients with a relevant benefit from adaptive pulmonary MRgSBRT are a cornerstone for the clinical introduction of this innovative approach.

The adaptation frequency observed in this cohort agrees with two previous reports on peripheral and central lung tumors, where >90% of adapted plans were considered advantageous, mostly due to improved PTV coverage (7, 15). Conversely, another analysis of MRgSBRT that comprised five cases of ULT found that plan adaptation was necessary in merely 40% of fractions, mostly due to OAR constraint violations (16). This discrepancy might be caused by the application of a more aggressive fractionation with homogenous dose prescription (16).

Plan adaptation improved the overall PTV coverage (+6.3%) and BED_min_ inside the PTV (+9 Gy), but PTV coverage was still deficient in several cases of ULT, which agrees with prior studies (7, 15, 16). Correspondingly, our LMM were compatible with a smaller gain in PTV coverage for larger PTV sizes and for ULT. This is probably attributable to the fact that most small and peripheral tumors lie far from OAR, which simplifies dose intensification to the target volumes. Moreover, the higher relative impact of dose improvements in smaller tumors should be considered. Conversely, ultracentral and potentially large lung tumors touch critical structures to a greater extent, so that PTV coverage literally collides with the prioritized OAR constraints.

The gains in PTV coverage and PTV BED_min_ translated to an increased BED_min_ inside the CTV (+6.5 Gy) and the GTV (+2.9 Gy), which is also supported by previous works (15). However, the BED_mean_, which seems to be the most important determinant of local tumor control after pulmonary SBRT (32), presented only a minor increase inside the PTV (+1.4 Gy) and CTV (+0.8 Gy) with a very weak trend inside the GTV (+0.6 Gy). In this context, it should be further discussed that the moderately prolonged dose delivery times due to gating might allow intrafractional tumor cell repopulation (33, 34). Previous works have suggested that dose delivery times >15–30 min might have a detrimental effect on SBRT efficacy (34–37). In this cohort, dose delivery times were in the range of 15 min and were shorter for ULT due to lower single doses. Hence, a potentially detrimental effect of the prolonged dose delivery time on treatment efficacy was probably small. Furthermore, high dose fractionations such as 3 × 15–20 Gy may be used in peripheral lung tumors far from sensitive OAR to achieve higher doses both inside the PTV and at the PTV borders. As a consequence, the moderate benefits in PTV coverage due to plan adaptation may become clinically meaningless. Here, the main advantage of MRgSBRT is probably gated dose delivery to abandon ITV concepts and spare healthy lung tissue. However, not all patients with peripheral lung tumors far from sensitive OAR qualify for high dose fractionation. Using higher single doses leads to considerably longer treatment sessions when using gated dose delivery and thus requires a certain pulmonary function capacity to follow breathing commands for a longer time. In fact, we often had to employ more protracted fractionations with lower single doses due to generally reduced pulmonary function and pulmonary comorbidity in this cohort. Finally, plan adaptation could also play a role in very high-dose single fraction SBRT, even though first data suggested limited clinical benefits (38).

All in all, plan adaptation yields moderate benefits in PTV coverage and doses, whose clinical relevance is disputable. Cases where more protracted, lower-dose fractionation is necessary, e.g., due to reduced pulmonary function or high-risk tumor location, could benefit from plan adaptation. Furthermore, it seems that plan adaptation is particularly useful in tumors close to vulnerable OAR, such as ULT, to allow careful dose intensification inside the PTV while minimizing violations of OAR dose constraints.

Our analysis demonstrated negligible PTV growth during MRgSBRT similar to previous investigations of this technique (7, 15), whereas a continuous decline in tumor size has been reported during normofractionated RT (39). This difference may be explained technically: SBRT applies higher single doses with possible initial swelling of the tumor and short treatment courses with insufficient observation time to detect the tumor response. Moreover, the small increase in PTV size that we found could reflect recontouring variability between different observers and also different imaging modalities rather than frank changes in tumor size.

We present the first dedicated analysis of PTV overdoses before and after MR-guided plan adaptation to our knowledge. PTV overdoses were present in 1 out of 5 predicted plans, which included, cases with spread out dose hotspots encompassing >5% of the PTV. For tumors whose PTV overlaps with a vulnerable OAR, especially ULT, such dose hotspots could reach the respective OAR and lead to a severe overdosage. This might explain the excessive toxicity in the prospective HILUS trial, where conventional non-adaptive SBRT techniques were used to treat ULT with maximum doses of 150% allowed inside the PTV (23). Furthermore, there is an exceptionally large heterogeneity of patient outcomes including fatal complications in retrospective series following non-adaptive SBRT of ULT (21, 22), which might be explained by varying SBRT techniques and thus varying occurrences of high dose areas as well. In this cohort, we chose a conservative approach and used plan adaptation to successfully reduce the number and size of dose hotspots independently from tumor location. Conversely, one might argue that the high precision of MRgSBRT is sufficient to safely escalate the PTV dose as long as OAR dose constraints are met. However, MRgSBRT still suffers from several uncertainties, particularly due to imaging artifacts, limitations of image registration and potentially unnoticed organ motion after plan adaptation. While the optimal approach to the maximum dose inside the PTV remains a matter of debate, the frequent occurrence of PTV overdoses generally supports the use of adaptive MRgSBRT for tumors in close proximity to sensitive OAR.

Plan adaptation significantly reduced the number of fractions with (non-lung) OAR constraint violations in agreement with previous reports (15, 16). The only remaining violation occurred in the case of an ULT, where OAR dose constraints could be met in all other fractions. Our statistical models were compatible with a strong reduction of OAR constraint violations due to plan adaptation but could not support an influence of ultracentral tumor location on violation of OAR constraints. On the other hand, descriptive analysis demonstrated that OAR constraint violations mostly occurred in tumors whose PTV already touched a critical structure in the initial plan. This included non-ultracentral tumors, in specific one central tumor with contact to the aorta and one peripheral tumor at the brachial plexus. Evidently, a high risk of OAR overdose is not restricted to ultracentral tumor location. Moreover, only one SBRT fraction with a risk-optimized fractionation of 10 × 5 Gy (BED_10_ = 75 Gy) applied to ULT presented OAR constraint violations inside the predicted plans. All the other OAR constraint violations occurred for more aggressive fractionations. Therefore, the highest benefit of plan adaptation should be expected in cases where tumor location is initially adjacent to a sensitive OAR and where application of more aggressive fractionations is intended. This explicitly includes tumors in non-ultracentral location, but ULT remain the highest risk category, which has recently been underpinned by the concerningly high morbidity (34% ≥grade 3 toxicity) and mortality (15% grade 5 toxicity) rates inside the HILUS trial (23). As a side note, our data supports a risk-optimized fractionation of 10 × 5 Gy to ULT (40) if MRgSBRT is not feasible because OAR dose violations mostly occurred for more aggressive fractionation schemes.

Based on our results, we currently prepare a clinical phase I dose escalation trial of adaptive MRgSBRT for ULT, the MAGELLAN trial, with the lowest dose level being 10 × 5 Gy.

Strengths of this analysis include the evaluation of the largest cohort of ULT treated with adaptive MRgSBRT so far, where ultracentral location was defined as an overlap of the PTV with the PBT, esophagus and/or pulmonary artery. Moreover, a dedicated analysis of benefits in different lung tumor locations was performed based on a Bayesian approach. Limitations of our study include that comparison of predicted and adapted plans was only possible for fractions where plan adaptation was performed (93%). The most common reason for not performing plan adaptation was that the treating physician expected only minor clinical improvements, so that our analysis could have overestimated the benefits of plan adaptation. Secondly, PTV changes over time due to recontouring on the MRI of the day might have confounded the results, but our analysis suggested only minor changes. Finally, different dose fractionations were applied due to different tumor locations and different clinical performance of patients.

## Conclusions

The use of adaptive MRgSBRT is highly recommendable for ablative treatment of lung tumors whose PTV touches a sensitive OAR in the baseline RT plan, which confers the highest risk for overdosing the respective OAR. Here, online plan adaptation minimizes overdoses to sensitive OAR while maintaining best-possible PTV coverage. More research is needed to determine the benefit of adaptive treatment in lung tumors that lie further away from sensitive OAR.

## Data Availability Statement

The datasets presented in this article are not readily available because according to the terms of the local ethics board approval underlying the prospective registries, the authors are not allowed to make the underlying patient data publicly available. Requests to access the datasets should be directed to juliane.hoerner-rieber@med.uni-heidelberg.de.

## Ethics Statement

The studies involving human participants were reviewed and approved by the Ethikkommission der Medizinischen Fakultät Heidelberg. The patients/participants provided their written informed consent to participate in this study.

## Author Contributions

Conceptualization: SR and JH-R. Data curation: SR, CB, FW, PH, TE, TH, JR, CR, LK, and SA. Formal analysis: SR. Methodology: SR, MP, and JH-R. Supervision: HW, MT, SA, JD, SK, and JH-R. Writing—original draft: SR and JH-R. Writing—review & editing: all authors. All authors contributed to the article and approved the submitted version.

## Funding

SR and TH are funded by the Physician-Scientist Program of Heidelberg University, Faculty of Medicine. The sponsor was not involved in any step of study design, analysis or publication.

## Conflict of Interest

JH-R and SK received speaker fees and travel reimbursement from ViewRay Inc. JH-R received travel reimbursement from IntraOP Medical and Elekta Instrument AB and a grant from IntraOP Medical outside the submitted work. SA received grants from Accuray International Sàrl, Merck Serono GmbH and Novocure GmbH outside the submitted work. SA received consulting fees from Accuray International Sàrl and honoraria for lectures/presentations from Accuray International Sàrl and MSD outside the submitted work. SA received travel reimbursements from AstraZeneca outside the submitted work. SA participated on advisory boards for Sanofi Genzyme outside the submitted work. JD received grants from CRI—The Clinical Research Institute GmbH, View Ray Inc., Accuray Incorporated, Accuray International Sàrl, RaySearch Laboratories AB, Vision RT limited, Astellas Pharma GmbH, Astra Zeneca GmbH, Solution Akademie GmbH, Ergomed PLC Surrey Research Park, Merck Serono GmbH, Siemens Healthcare GmbH, Quintiles GmbH, Pharmaceutical Research Associates GmbH, Boehringer Ingelheim Pharma GmbH Co, PTW-Freiburg Dr. Pychlau GmbH, Nanobiotix A.A. and IntraOP Medical outside the submitted work. MT received honoraria for lectures/presentations from AbbVie, AstraZeneca, Bristol-Myers Squibb, Boehringer-Ingelheim, Celgene, Chugai, Janssen, Lilly, MSD, Novartis, Pfizer, Roche and Takeda outside the submitted work. MT received travel reimbursement from AbbVie, Amgen, AstraZeneca, Bristol-Myers Squibb, Boehringer Ingelheim, Celgene, Chugai Pharma, Janssen, Lilly, Merck, MSD, Novartis, Pfizer, Roche and Takeda outside the submitted work. MT received research grants from Bristol-Myers Squibb, Astrazeneca, Roche and Takeda outside the submitted work. MT participated on advisory boards for AbbVie, Amgen, AstraZeneca, Bristol-Myers Squibb, Boehringer Ingelheim, Celgene, Chugai Pharma, Janssen, Lilly, Merck, MSD, Novartis, Pfizer, Roche and Takeda outside the submitted work. TE received grants from Ruprecht-Karls Universität Heidelberg, Herbert Kienzle Foundation and Else Kröner-Fresenius Foundation and received travel reimbursement from Bristol-Myers Squibb outside the submitted work.

The remaining authors declare that the research was conducted in the absence of any commercial or financial relationships that could be construed as a potential conflict of interest.

## Publisher’s Note

All claims expressed in this article are solely those of the authors and do not necessarily represent those of their affiliated organizations, or those of the publisher, the editors and the reviewers. Any product that may be evaluated in this article, or claim that may be made by its manufacturer, is not guaranteed or endorsed by the publisher.
